# The absence of the leukotriene B_4_ receptor BLT1 attenuates peripheral inflammation and spinal nociceptive processing following intraplantar formalin injury

**DOI:** 10.1186/s12990-015-0010-9

**Published:** 2015-03-12

**Authors:** Miho Asahara, Nobuko Ito, Takehiko Yokomizo, Motonao Nakamura, Takao Shimizu, Yoshitsugu Yamada

**Affiliations:** Department of Anesthesiology, Faculty of Medicine, The University of Tokyo, Tokyo, Japan; Department of Biochemistry, Juntendo University School of Medicine, Tokyo, Japan; Department of Life Science, Faculty of Science, Okayama University of Science, Okayama, Japan; Department of Lipid Signaling Project, Research Institute, National Center for Global Health and Medicine, Tokyo, Japan; Department of Lipidomics, Faculty of Medicine, The University of Tokyo, Tokyo, Japan

**Keywords:** Inflammation, Leukotriene, Formalin test, Sensitization

## Abstract

**Background:**

Leukotriene B_4_ (LTB_4_) is a potent lipid mediator of inflammation, and its biological effects are mediated primarily through the high affinity LTB_4_ receptor BLT1. Although numerous studies have reported that LTB_4_-BLT1 signaling is involved in inflammatory diseases, the role of BLT1 signaling in pain remains undefined. To clarify the role of LTB_4_-BLT1 signaling in acute inflammatory pain induced by tissue injury, we performed pain behavioral analysis and assessment of local inflammation induced by peripheral formalin injections in BLT1 knockout mice. We examined the phosphorylation of cAMP response element-binding protein (CREB) in the spinal cord both in wild-type and BLT1 knockout mice because phosphorylation of CREB in spinal cord neurons is important for nociceptive sensitization following peripheral injury. We also examined the effect of a BLT1 antagonist on formalin-induced pain responses in mice.

**Results:**

BLT1 knockout mice exhibited markedly attenuated nociceptive responses induced by intraplantar formalin injections. Edema formation and neutrophil infiltration in the paw were significantly decreased in BLT1 knockout mice compared with wild-type mice. Phosphorylation of CREB in the spinal cord after the intraplantar formalin injection was decreased in BLT1 knockout mice. In addition, mice pretreated with a BLT1 antagonist showed reduced nociception and attenuated CREB phosphorylation in the spinal cord after the formalin injection.

**Conclusions:**

Our data suggest that LTB_4_-BLT1 axis contributes not only to the peripheral inflammation but also to the neuronal activation in the spinal cord induced by intraplantar formalin injections. Thus, LTB_4_-BLT1 signaling is a potential target for therapeutic intervention of acute and persistent pain induced by tissue injury.

## Background

Leukotriene B_4_ (LTB_4_; 5(S),12(R),-dihydroxy-6,14-cis-8,10-trans-eicosatetraenoic acid), a metabolite of arachidonic acid catalyzed by 5-lipoxygenase and leukotriene A_4_ hydrolase, is a potent lipid chemoattractant responsible for recruitment of inflammatory cells to inflammatory sites [1]. The potent biological effects of LTB_4_ are mediated primarily through a high affinity interaction with the G-protein-coupled receptor termed LTB_4_ receptor type 1 (BLT1) [2]. BLT1 is expressed in a variety of cells, including macrophages and their precursors, monocytes, as well as neutrophils, differentiated T cells and osteoclasts [3,4]. We and others established BLT1 knockout mice to clarify the physiological and pathophysiological roles of LTB_4_-BLT1 signaling *in vivo* [5-7]. Previous studies using BLT1 knockout mice showed that LTB_4_-BLT1 signaling is strongly related to a variety of immune responses and inflammatory diseases, including bronchial asthma [7,8], multiple sclerosis [9], rheumatoid arthritis [10] and psoriasis [11].

Several studies have elucidated the role of the LTB_4_-BLT1 axis in modulating pain signals. Local injections of LTB_4_ cause both thermal and mechanical hyperalgesia [12]. Intrathecal injections of LTB_4_ augment the nociceptive responses after intraplantar injections of formalin, and these responses are suppressed by a 5-lipoxygenase inhibitor or a BLT1 antagonist [13]. The expression of BLT1 in the rat dorsal root ganglion (DRG) and spinal cord was confirmed by *in situ* hybridization, and BLT1 mRNA in spinal cord neurons increased in the rat spared nerve injury model of neuropathic pain [14,15]. The expression of leukotriene A_4_ hydrolase, an enzyme to produce LTB_4_, was confirmed in the sensory nervous system, including in lamina II of the spinal cord [16]. Although these studies suggest that the LTB_4_-BLT1 system is involved in nociception, little is known about the endogenous LTB_4_-BLT1 axis in nociception.

Peripheral tissue injury causes the release of various mediators from damaged and inflammatory cells, which in turn activate and sensitize primary sensory neurons to produce persistent pain [17,18]. These peripheral changes induce the release of some neurotransmitters in the spinal cord and activate intracellular protein kinases, such as protein kinase A, protein kinase C, Ca^2+^/calmodulin-dependent kinases and mitogen activated kinases, leading to a change in gene expression through cyclic AMP response element-binding protein (CREB), which triggers the activation of the pain pathway [19-21]. CREB is activated by phosphorylation of serine 133 in dorsal horn neurons [20] and is involved in pain processing, for example, at the level of the spinal cord as was observed in a study of tissue injury-induced inflammation and hyperalgesia following intraplantar injections of formalin [17,22,23].

In the present study, we investigated the role of the LTB_4_-BLT1 axis in the persistent pain behavior caused by tissue injury in BLT1 knockout mice and examined whether deletion of BLT1 resulted in suppression of CREB activation in the spinal cord. We also studied the anti-nociceptive efficacy induced by blocking BLT1 and the potential site of the BLT1 action using a BLT1 antagonist.

## Results

### Reduced formalin-induced pain behaviors in BLT1 knockout mice

**Figure 1:**
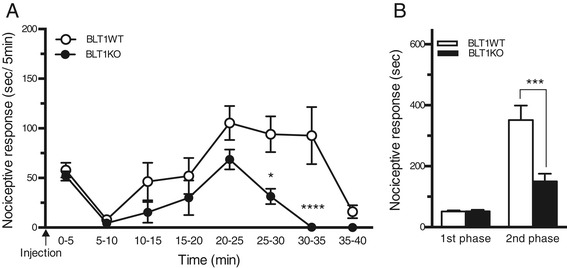
**Attenuation of formalin-induced pain behavior in LTB**_**4**_ **receptor type 1 knockout (BLT1KO) mice. (A)** Time course of pain behaviors after formalin injection (* p < 0.05, **** p < 0.0001 vs. BLT1-wild-type (BLT1WT) mice). **(B)** Total duration of pain behaviors during the 1st (0–10 min) and the 2nd phases (11–40 min) (*** p < 0.001 vs. WT mice, n = 5, a two-way ANOVA with a Bonferroni post hoc test).

To evaluate the tissue injury-induced acute nociceptive response in BLT1 knockout (BLT1KO) mice, we performed the formalin test. Intraplantar injections of formalin produced a typical biphasic pain response during a 40 min observational period (first phase, 0–10 min after formalin injection; second phase, 11–40 min after formalin injection) in BLT1WT as well as BLT1KO mice (Figure 1A). The time spent licking, biting and flinching was compared at every 5 min interval and no significant differences were observed for up to 25 min after the formalin injection (Figure 1A). However, from 25 to 35 min after the formalin injection, a significant difference between the WT and BLT1 KO mice was observed (p < 0.05). During the first phase, both BLT1WT and BLT1KO mice spent equal amounts of time performing nociceptive responses (Figure 1B). However, in the second phase, the formalin-induced pain behavior was significantly attenuated in BLT1 KO mice compared with that in WT mice (p < 0.001) (Figure 1B). In naïve BLT1KO mice, thermal and mechanical responses were examined by application of radiant heat (Ugo Basil, Italy) or various weights of von-Frey filaments (Stoelting, Wood Dale, IL, USA) to the hind paw and the withdrawal latencies were calculated. Both the calculated thermal and mechanical responses were identical to those observed in the BLT1WT littermates (data not shown).

### Characterization of DRG neurons in BLT1 knockout mice

**Figure 2:**
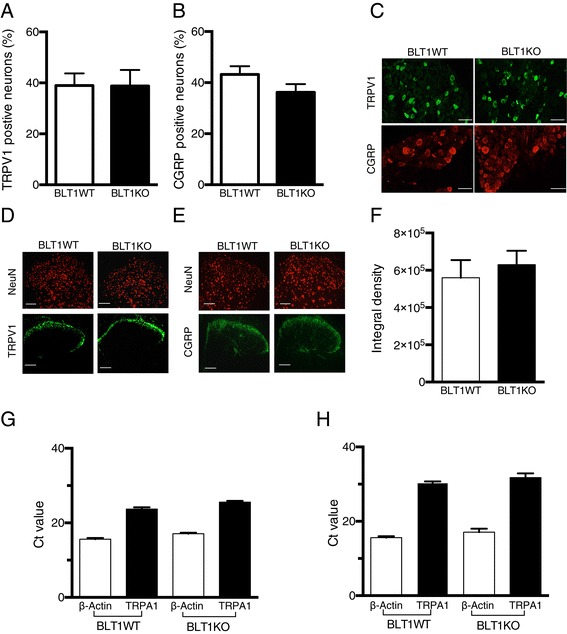
**TRPV1 and CGRP expression profiles of nociceptive neurons are unaffected by BLT1 receptor knockout (BLT1KO). (A)** Transient receptor potential vanilloid 1(TRPV1)- and **(B)** calcitonin-gene related peptide (CGRP)-positive neurons in the L4 dorsal root ganglion (DRG). **(C)** Representative immunofluorescence images show the localization of TRPV1 (green) and CGRP (red) in L4 DRG neurons (magnification, 200×). Scale bar represents 50 μm. **(D, E)** Representative immunofluorescence images showing the localization of TRPV1 (green), CGRP (green) and NeuN (red) in the lumbar spinal cord (magnification, 100×). Scale bars represent 100 μm. **(F)** Quantitative analysis of TRPV1-immunopositive density. β-Actin and TRPA1 mRNA levels in the DRG **(G)** and spinal cord **(H)** analyzed by quantitative RT-PCR. (n = 5–7, a Kruskal-Wallis with Dunn’s multiple comparison test ). Data was expressed as Ct value.

To evaluate the difference in the properties of nociceptive neurons between BLT1WT and BLT1KO mice, we analyzed the expression of the transient receptor potential vanilloid 1 (TRPV1) receptor as a marker of noxious heat sensor and CGRP as a marker of peptidergic nociceptive neurons in naïve DRG neurons using immunohistochemistry. As shown in Figure 2AB and C no significant differences were observed in the percent of the TRPV1-positive neurons and CGRP-positive neurons between BLT1WT and BLT1KO mice (39.0 ± 4.8% vs. 38.8 ± 6.3% for TRPV1 and 43.2 ± 3.2% vs. 36.2 ± 3.2% for CGRP). In addition, immunoreactivity for TRPV1 and CGRP in the dorsal horn was observed in the superficial lamina for both WT and BLT1KO mice (Figure 2D and E). No significant differences were observed in integral densities of TRPV1 immunoreactivity between genotypes (Figure 2F). Because no good antibodies are currently available for transient receptor potential ankyrin 1 (TRPA1), we confirmed the expression of TRPA1 in the lumbar DRG and spinal cord using quantitative RT-PCR analysis. Since several reports indicated the function of spinal TRPA1 [24-27], we analyzed and compared the expression of TRPA1 in spinal cord. There were no significant differences between the genotypes (Figure 2G, and H). Ct values of spinal cord (30.4±0.4 in WT, 31.8±1.1 in BLT1KO) were higher than Ct values of DRG (23.7±0.5 in WT, 25.6±0.3 in BLT1 KO).

These results suggest that the properties of nociceptive neurons in BLT1KO mice were similar to those in BLT1WT mice.

### Reduced peripheral inflammation in BLT1 knockout mice

**Figure 3:**
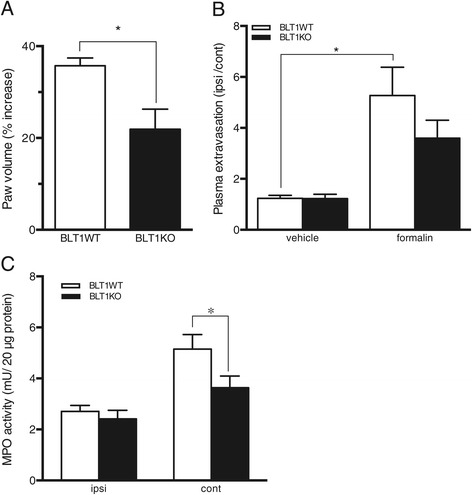
**Local inflammation induced by formalin injections is attenuated in BLT1KO mice. (A)** Paw edema formation 1 h after formalin injection. (* p < 0.05 vs. BLT1WT mice, n = 5, unpaired Student’s t-test with Welch’s correction). **(B)** Quantification of Evans blue dye extravasation 1 h after formalin or vehicle injection. (p = 0.2366 vs. BLT1WT mice, n = 5-7, * p < 0.05 vs. vehicle, n = 5–7, Kruskal-Wallis with Dunn’s multiple comparison test). **(C)** Myeloperoxidase (MPO) activity in the paw 1 h after formalin injection. (* p < 0.05 vs. BLT1WT mice, n = 8–9, two-way ANOVA with a Bonferroni post hoc test).

We next analyzed local inflammatory changes 1 h after formalin injections in mice with both genotypes because the second phase is involved in the hyperexcitability of primary afferent neurons in response to inflammation [28]. LTB_4_-BLT1 signaling is strongly related to inflammatory diseases and conditions [29], and therefore, we hypothesized that the peripheral inflammation following formalin injections would be reduced in BLT1KO mice. To assess local inflammation, we measured the degree of formalin-induced swelling, local plasma extravasation and myeloperoxidase (MPO) activities. The degree of formalin-induced swelling was analyzed by measuring the paw volume. In BLT1KO mice, the percent increase in paw volume following a formalin injection was significantly lower than that in BLT1WT mice (p < 0.05) (Figure 3A). An increase in plasma extravasation was observed in the formalin-injected paw compared with that in the vehicle-injected paw in mice with both genotypes; however, the plasma extravasation was significantly increased only in BLT1WT mice (p < 0.05) and the degree of plasma extravasation following a formalin injection was not significantly different between BLT1KO and BLT1WT mice. (p = 0.2366) (Figure 3B). We also measured MPO activity in paw tissue homogenates, comparing the results between the formalin-injected and contralateral sides. MPO is expressed in myeloid and monocytic cells and is an indicator of polymorphonuclear neutrophil infiltration. In BLT1KO mice, MPO activity on the formalin-injected side was significantly lower than that in the BLT1WT mice (p < 0.05) (Figure 3C). These results reveal that the absence of LTB_4_-BLT1 signaling attenuates peripheral edema formation and inflammation.

### Reduced CREB phosphorylation in spinal cord neurons following intraplantar formalin injection in BLT1 knockout mice

**Figure 4:**
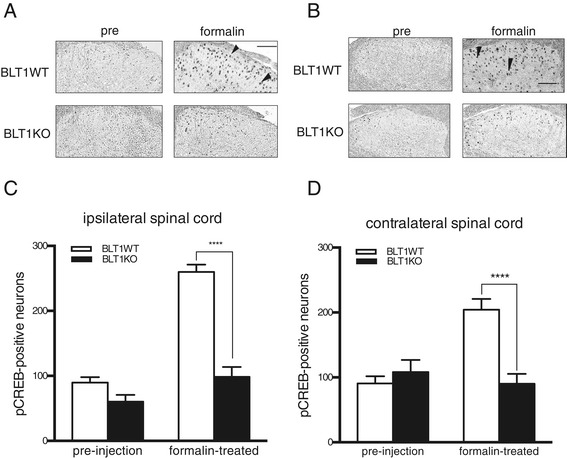
**The expression of phosphorylated CREB (pCREB) in the dorsal horn is attenuated in BLT1KO mice.** Representative diaminobenzidine-stained images showing pCREB-positive neurons in sections of the ipsilateral **(A)** and contralateral **(B)** dorsal horn of the lumbar spinal cord before or 20 min after a peripheral formalin injection (magnification, 100×). Arrowheads indicate pCREB-positive neurons. The number of pCREB-positive neurons in the ipsilateral **(C)** and contralateral **(D)** dorsal horn of the lumbar spinal cord prior to or 20 min after formalin injections. (**** p < 0.0001 vs. BLT1WT mice, n = 6, two-way ANOVA with Bonferroni post hoc test).

To investigate whether LTB_4_-BLT1 signaling is involved in the spinal modulation of nociceptive sensitization following peripheral formalin injections, we analyzed the activation of CREB in the dorsal horn of the spinal cord in BLT1KO mice. CREB is phosphorylated at serine 133 by various kinases, such as extracellular signal-regulated kinase (ERK), and induces transcription of immediate early genes, such as c-Fos, COX2 and TrkB. This cascade is crucial for sensitizing sensory neurons, leading to persistent pain hyperalgesia [17]. We detected phosphorylated CREB (pCREB) in the bilateral dorsal horn of the spinal cord following formalin injections into the hind paw (Figure 4A and B). The number of pCREB-immunoreactive neurons in BLT1KO mice after the formalin injection was significantly lower than that in BLT1WT mice in both the ipsilateral and contralateral dorsal horns (p < 0.05) (Figure 4C and D). These results suggest that LTB_4_-BLT1 signaling is required for the activation of CREB in the dorsal horn following formalin injections and contributes to the modulation of sensitization.

### Pretreatment with the BLT1 antagonist ONO-4057 reduced formalin-induced pain behavior in mice

**Figure 5:**
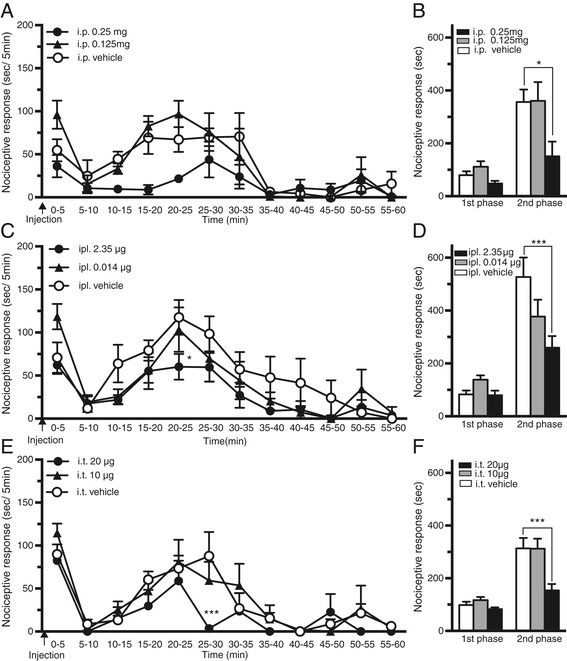
**Effects of ONO-4057 on pain behavior in the formalin test.** Time course of pain behavior in mice pretreated with intraperitoneal (i.p.) **(A)**, intraplantar (ipl.) **(C)** or intrathecal (i.t.) **(E)** injections of ONO-4057 (* p < 0.05, *** p < 0.001 vs. vehicle). Total duration of pain behaviors in mice pretreated with i.p. **(B)**, ipl. **(D)** and i.t. **(F)** injections of ONO-4057. (* p < 0.05, *** p < 0.001 vs. vehicle; n = 5–7; two-way ANOVA with Bonferroni post hoc tests).

The therapeutic efficacy of blocking LTB_4_-BLT1 signaling in formalin-injected mice was examined by pretreating mice with the BLT1 antagonist ONO-4057. To determine the potential sites of the LTB_4_-BLT1 signaling actions, we examined three treatment routes: intraperitoneal (i.p.), intraplantar (ipl.) and intrathecal (i.t.). In the first phase, the duration of the nociceptive responses was similar for all mice irrespective of treatment route and was not significantly different from that in the vehicle-treated group (Figure 5A,C and E). However, in the second phase, the duration of the nociceptive responses was significantly reduced for every treatment route compared with that in the vehicle-treated mice (p < 0.05) (Figure 5B,D and F). These results indicate that LTB_4_-BLT1 signaling is involved in nociceptive responses not only at peripheral sites, but also in the spinal cord.

### ONO-4057 treatment reduced peripheral inflammation and CREB phosphorylation in spinal cord neurons following intraplantar formalin injections in mice

**Figure 6:**
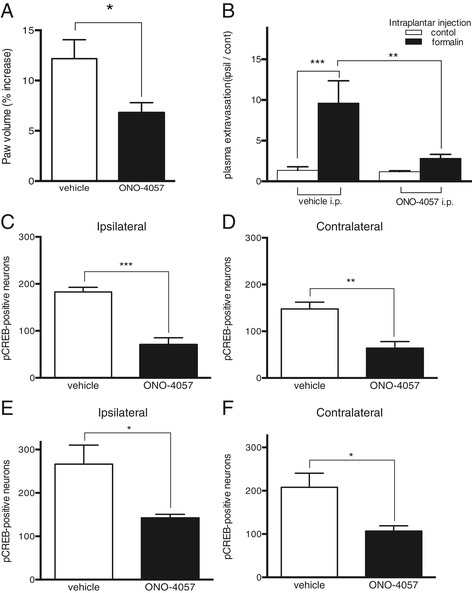
**ONO-4057 pretreatment affects peripheral inflammation and pCREB expression in the dorsal horn 20 min after intraplantar formalin injection. (A)** Paw edema formation 1 h after formalin injection. (* p < 0.05, ONO-4057 (i.p. 0.25 mg) treated mice vs. vehicle (i.p.) treated mice, n = 5–6, unpaired Student’s t-test with Welch’s correction). **(B)** Quantification of Evans blue dye extravasation 1 h after formalin or vehicle injection. (** p < 0.01,*** p < 0.001, n = 6, Kruskal-Wallis with Dunn’s multiple comparison test). The number of phosphorylated CREB (pCREB)-positive neurons in the ipsilateral **(C)** and contralateral **(D)** dorsal horns of the spinal cord after intraplantar injections of formalin in mice pretreated with intraperitoneal ONO-4057 (0.25 mg, i.p.). (** p < 0.01, *** p < 0.001 vs. vehicle-pretreated mice, n = 4–5, unpaired Student’s t-test with Welch’s correction). Counts of pCREB-positive neurons in the ipsilateral **(E)** and contralateral **(F)** dorsal horn of the spinal cord after intraplantar formalin injection in mice pretreated with intraplantar ONO-4057 (2.35 μg, ipl.) injections. (* p < 0.05 vs. vehicle-pretreated mice, n = 5, unpaired Student’s t-test with Welch’s correction).

Next, we examined whether blockade of BLT1 signaling reduced peripheral inflammation. In BLT1 antagonist pretreatment group, increase of paw volume (p < 0.05) and plasma extravasation (p < 0.001) was significantly lower than that of vehicle treatment group (Figure 6A,B). We examined whether peripheral or systemic blockade of BLT1 signaling suppressed pCREB expression in the dorsal horn of the spinal cord after intraplantar formalin injection. The number of pCREB-immunoreactive neurons in the dorsal horn of mice pretreated with ONO-4057 via the intraperitoneal (Figure 6C and D) or intraplantar route (Figure 6E and F) after the formalin injection was significantly lower than that in vehicle-treated mice (p < 0.05). These results indicate that either peripheral or systemic blockade of LTB_4_-BLT1 signaling suppresses CREB activation in the dorsal horn and attenuates sensitization.

## Discussion

We studied mice genetically lacking BLT1 in the formalin test, which is commonly used to assess acute pain (first phase) and the subsequent inflammatory pain (second phase) induced by tissue injury. We found no alteration in pain behavior in the first phase, but the nociceptive behavior in the second phase was significantly attenuated in BLT1KO mice (Figure 1). The second phase of the formalin test represents the combined effects of afferent input from the peripheral response and sensitization in the dorsal horn [28]. In the present study, following formalin injections, the peripheral inflammatory responses were reduced in BLT1KO mice, attenuating the nociceptive responses in the second phase. Furthermore, activation of CREB in the dorsal horn, which triggers activity-dependent transcriptional changes and sensitization, was reduced in BLT1KO mice following formalin injections. This suppression of the functional changes in the dorsal horn may also lead to the attenuation of nociceptive responses in second phase. These results indicate that LTB_4_-BLT1 signaling is a crucial element in central sensitization as well as in the peripheral inflammatory response.

LTB_4_ plays an important role in the activation of inflammatory cells and its acute reaction is initiated by its binding to the high affinity receptor BLT1. Previous studies using BLT1KO mice or BLT1 antagonists revealed that BLT1 blockade dramatically inhibits neutrophil infiltration in several inflammatory diseases, such as rheumatoid arthritis, peritonitis and psoriasis, resulting in amelioration of the disease condition [6,10,11,30]. LTB_4_-BLT1 signaling is also known to enhance vascular permeability through activation of polymorphonuclear neutrophils [31]. In the present study, we observed reduced local swelling, extravasation and MPO activity in the paw following intraplantar formalin injections in BLT1KO mice that were consistent with a role of LTB_4_-BLT1 signaling for the progression of the inflammation. These findings confirm the hypothesis that LTB_4_-BLT1 signaling contributes substantially to peripheral inflammatory responses and induces nociceptive responses to tissue injury-induced pain.

TRPV1 and TRPA1 belong to the transient receptor potential (TRP) ion channel superfamily, which are expressed in primary afferent nociceptors and strongly implicated in the genesis of inflammatory pain [32,33]. Activation of TRPV1 and TRPA1 enhance inflammatory pain not only via peripheral sensitization [32,34,35] but also via central sensitization by increasing glutamate release from primary afferent terminals to enhance synaptic transmission [24]. It was reported that the formalin-induced effects on nociceptors were mainly attributed to TRPA1 [36]. On the other hand, Shields et al. reported that formalin-induced pain behaviors persisted after the ablation of C-fiber nociceptors that express these TRP channels and suggested an involvement of all types of afferent nociceptors [37]. We confirmed the expression of TRPA1 and TRPV1 in the DRG of naïve BLT1KO mice, and also confirmed the expression of CGRP as a marker of peptidergic nociceptive neurons in the DRG of naïve BLT1 KO mice. These results indicated that the expression of afferent nociceptors in BLT1KO mice was not related to the reduction of formalin-induced pain responses. Several lipid mediators including LTB_4_ have been identified as endogenous activators of TRP channels [38-40]. Among these, LTB_4_ is an especially attractive candidate as an endogenous ligand of TRPV1 in inflammatory states; however, micromolar concentrations of lipid mediators are required to activate TRP channels [38]. Therefore, whether LTB_4_ is an endogenous ligand for TRPV1 remains controversial. Modulation of the TRPV1 current by several G-protein-coupled receptors, such as the prostanoid receptors EP4 [41], EP1 and IP [42], suggested that BLT1 might also modulate TRPV1 function for enhancement of sensitization. Inflammatory mediators such as ATP, bradykinin and tryptase potentiate TRPV1 activity in a protein kinase C-dependent [43-45] or protein kinase A-dependent manner [46,47] in DRG neurons. Andoh et al. reported that LTB_4_ induced Ca^2+^ increases in several TRPV1-positive DRG neurons using Ca^2+^ imaging [48], indicating that the presence of functional BLT1 receptors in nociceptive neurons and LTB_4_-BLT1 signaling modulated the activation of the TRPV1 channel. It is known that inflammatory mediators, lipoxygenase metabolites and superoxide released from PMNLs through LTB_4_-BLT1 signaling immediately could activate primary afferent fibers directly [49]. Considering that nociceptive responses were observed quite rapidly (within 30 min) after formalin injection, there is a possibility that BLT1 is expressed in primary afferent fibers. It is plausible that the LTB_4_ produced shortly after tissue injury activated primary afferent fibers that express both BLT1 and TRPV1 channels. Further studies including the expression of BLT1 in primary afferent fibers will be required to clarify the downstream signaling of LTB_4_-BLT1 that potentiates TRPV1 activity.

TRPA1 is also capable of mediating acute and inflammatory pain and several reports indicated the crosstalk between GPCR signaling and TRPA1 modulation [35,50,51]. Wang et. al reported that bradykinin sensitize TRPA1 via activation of PLC [51] indicating that downstream signaling of LTB_4_-BLT1 might sensitize TRPA1.Considering that formalin is one of the TRPA1 agonists [36], blocking of LTB_4_-BLT1 signaling might affect the sensitization of TRPA1 in formalin test. Heterologous expression system using either or both BLT1 and TRPA1 expressed vectors is needed to clarify the interaction of these two receptor.

We tried to identify the potential site of BLT1 signaling following formalin injection using BLT1 antagonist via three routes (intraperitoneal, intraplantar and intrathecal route) and found that nociceptive responses were significantly reduced in every three-group compare with vehicle treatment group. Attenuation of nociceptive responses in intraplantar pretreatment group indicated that peripheral inflammation induced by a classical function of LTB_4_-BLT1 system contribute to the nociceptive responses following formalin injection. Attenuation of nociceptive responses was also observed in intrathecal pretreatment group, indicating that the existence of functional LTB_4_-BLT1 system that affects the progress of pain transmission in spinal cord neuron.

An important finding of the present study is that the lack of the LTB_4_-BLT1 system was associated with the suppressed activation of CREB in spinal cord neurons and consequent transcriptional modifications that induced persistent hypersensitivity. CREB activation in the spinal cord is induced by ERK activation [17]. The LTB_4_-BLT1 system may be associated with the ERK-CREB cascade either through peripheral inflammation, a classical function of the LTB_4_-BLT1 system that stimulates nociceptive primary afferent fibers and induces ERK activation of spinal cord neurons, or through direct ERK activation and subsequent CREB activation mediated by BLT1 in spinal cord neurons. Since ERK-CREB pathway is a series of sequential steps following formalin injection, [17] ERK activation would be expected to be reduced by blocking of LTB_4_-BLT1 signaling. ERK is activated by LTB_4_ through BLT1 in various cells [52-54] and there is one possibility that ERK is activated directly through LTB_4_-BLT1 signaling in neuron of dorsal horn. Interestingly, formalin-induced nociceptive responses during the second phase were significantly reduced by pretreatment with the BLT1 antagonist compared with vehicle pretreatment following not only intraplantar but also intrathecal injections. These results support our hypothesis that BLT1 activates the ERK-CREB cascade in spinal cord neurons. Stronger nociceptive responses and more pCREB-positive neurons following formalin injection were observed in mice with intraplantar injection of BLT1 antagonist. This is possibly due to the procedure of intraplantar injection itself. There are no significance among three treatment groups (p = 0.0951 vs. intraperitoneal group and p = 0.2589 vs. intrathecal group) in nociceptive response. CREB-binding sites have been found in the promoter regions of c-fos [55] and c-fos induction following CREB activation in the spinal cord is also expected to be reduced by the blockade of LTB_4_-BLT1 signaling.

Okubo et al. reported that BLT1 mRNA was upregulated in neurons of the dorsal horn and that an intrathecally administered BLT1 antagonist or 5- lipoxygenase inhibitor reduced allodynia after peripheral nerve injury [15]. These results support our data showing that LTB_4_-BLT1 signaling is involved in the progression of nociceptive responses at the level of spinal cord. LTB_4_-BLT1 signaling may also directly modulate the synaptic current in spinal dorsal horn neurons, generating functional consequences during central sensitization. Among the lipid mediators, prostaglandin E_2_ induced excitatory post-synaptic currents through the EP2 receptor [56], whereas platelet-activating factor did not alter the excitatory post-synaptic currents in dorsal horn neurons [57]. Further electrophysiological studies will be required to elucidate the direct modulation of excitatory synaptic current through the LTB_4_-BLT1 system.

Peripheral inflammation also activates dorsal horn astrocytes, and blockade of glial cell functions reduce the formalin-induced second phase nociceptive responses [58,59]. We speculate that LTB_4_ may be released from glial cells in a manner similar to that for the prostaglandins and inflammatory cytokines, bind to its specific receptor BLT1 on spinal cord neurons and initiate the cascade of sensitization, that is, the activation of ERK leading to the phosphorylation of CREB. However, the mechanisms underpinning LTB_4_-BLT1 signaling, including the physiology of this axis, in central terminals of the spinal cord requires additional examination.

## Conclusions

In conclusion, we found that the disruption of BLT1 gene in mice reduced formalin-induced pain behaviors, peripheral inflammation and activation of CREB in the dorsal horn of the spinal cord. In addition, pretreatment with a BLT1 antagonist was effective against formalin-induced pain behaviors. These results suggest that LTB_4_-BLT1 signaling may contribute not only to peripheral inflammation but also to the sensitization of nociceptors during persistent pain. Thus, LTB_4_-BLT1 signaling is a key component in pain mechanisms and a potential target for therapeutic intervention in acute and persistent pain.

## Methods

### Animals

BLT1KO mice were established using a conventional strategy [7], and 10–14-week-old BLT1KO mice and their wild-type littermates (C57BL/6 background) (body weights, 23–30 g) were used. The mice were housed under standard conditions (12 h light–dark cycle; lights on at 6:00 am) with free access to food and water. The experiments were approved by the ethics committee for the animal experiments of the University of Tokyo and performed according to the University of Tokyo’s guidelines for the care and use of laboratory animals.

### Formalin test

The formalin test was conducted during the light phase of the cycle. Each mouse was habituated in an individual observation cage for at least 30 min prior to an injection of 5% formalin (10 μL) into the dorsal surface of the right hind paw using a 30-gauge needle fitted to a microsyringe. After the injection, each mouse was immediately placed into a transparent observation cage. The time spent licking, biting and flinching the injected paw was recorded using a stopwatch in 5 min intervals for 40 or 60 min.

### Measurement of paw edema

The hind paw of a mouse was submerged to the ankle hairline within a plethysmometer (Muromachi Kikai Co. Tokyo, Japan), yielding a measure of paw volume. The measurements were performed before and 1 h after an intraplantar injection of formalin into the right hind paw. The increase in the paw volume was calculated using the baseline (before the injection) value according to the following formula: Increase in paw volume (%) = (paw volume value 1 h after formalin injection – paw volume value at baseline)/paw volume value at baseline.

### Vascular permeability assay

The vascular permeability assays were performed as described previously [60]. Briefly, mice were injected intraperitoneally (i.p.) with Evans Blue dye (80 mg/kg, Sigma-Aldrich, St. Louis, MO, USA, diluted in 100 μL of phosphate-buffered saline, PBS). Five minutes later, 10 μL of 5% formalin or vehicle (PBS) was intraplantarly injected. One hour after the injection, mice were deeply anesthetized with sodium pentobarbital (100 mg/kg, i.p.) and perfused with 30 mL of PBS. Both hind paws were removed. The samples were dried to remove excess liquid, weighed and incubated in 1 mL of dimethylformamide overnight at 52°C. After centrifugation at 10,000 × g for 20 min at 4°C, the optical density at a wavelength of 620 nm was measured with a spectrophotometer. Plasma extravasation was calculated as the concentration of Evans blue in the ipsilateral paw divided by that in the contralateral paw.

### MPO activity measurements

Paw skin tissues were removed 1 h after formalin injections, washed in PBS, frozen in liquid nitrogen and stored at −80°C until use. Samples were homogenized in 50 mM potassium phosphate buffer (KPB) solution (pH 7.4), subjected to three freeze-thaw cycles and centrifuged at 14,000 rpm at 4°C for 10 min. The supernatants were reacted with 100 μL of KPB (50 mM; pH 6.0) containing 0.157 mg/mL of O-dianisidine dihydrochloride (Sigma-Aldrich) and 0.0005% hydrogen peroxide. The absorbance was measured spectrophotometrically at a wavelength of 450 nm as described previously [61]. The MPO activity in each sample was calculated using a standard curve of commercially available MPO (Calbiochem, San Diego, CA, USA) and expressed in milliunits per 20 μg of protein in the supernatant samples.

### RNA extraction and qRT-PCR

Lumbar spinal cord and L1–6 DRGs were dissected and collected from naïve male wild-type (WT) and BLT1KO mice. Total RNA was extracted from spinal cord using Isogen (Nippon Gene Co. Tokyo, Japan) and from DRG using the Rneasy mini kit (QIAGEN, Hilden, Germany). The RNA (0.5 μg) was reversely transcribed to cDNA in 20 μL of a reaction mixture containing 10 mM deoxyribonucleotide triphosphates, 0.1 M dithiothreitol, 50 ng of oligo(dT) primer and 50 U of SuperScript II reverse transcriptase (all components purchased from Invitrogen, Carlsbad, CA, USA) for 52 min at 42°C.

Quantitative RT-PCR was performed with SYBRGreen (Applied Biosystems, Carlsbad, CA, USA) using a 7500 Real Time PCR system (Applied Biosystems) in 20 μL of reaction mixture. The mixture contained 5 μL of diluted RT-PCR product, 0.5 μM of each of the paired primers and 4 μL of real-time PCR SYBR Green Master Mix (Applied Biosystems). The PCR conditions were as follows: 50°C for 2 min and 95°C for 10 min, followed by 40 cycles at 95°C for 15 s; 60°C for 50 s; 72°C for 20 s. The qRT-PCR for the housekeeping gene β-actin was performed for each sample. The following primers were used for qRT-PCR: TRPA1, 5’-CCATGACCTGGCAGAATACC-3’ (forward) and 5’-TGGAGAGCGTCCTTCAGAAT-3’ (reverse) [62]; β-actin, 5’-ACCCACACTGTGCCCATCTA-3’ (forward) and 5’-GCCACAGGATTCCATACCCA-3’ (reverse). The relative mRNA level of the TRPA1 and β-actin were calculated from a standard curve. Data was represented as the mean Ct value in each sample.

### Immunohistochemistry

The mice were sacrificed and perfused transcardially with 30 mL of PBS followed by 30 mL of 4% paraformaldehyde. Lumbar enlargements of the spinal cord and L4 DRGs were removed and post-fixed in 4% paraformaldehyde overnight at 4°C, placed in 20% sucrose solution overnight at 4°C and embedded in O.C.T. compound (Sakura Finetek, Tokyo, Japan). Slices (10 μm thick) were prepared using a cryostat (Leica CM 1850, Wetzlar, Germany).

For immunohistochemical analysis of TRPV1, Neuronal Nuclei (NeuN) and calcitonin-gene related peptide (CGRP), spinal cords and DRGs from naïve BLT1WT and BLT1KO mice were used. The sections were incubated in 1% normal goat serum in PBS with 0.3% Triton X-100 for 1 h. Then, the sections were incubated with rabbit anti-mouse TRPV1 antibody (1:300, ACC-030, Alomone Labs, Jerusalem, Israel), rabbit anti-CGRP antibody (1:5000, C8198, Sigma) and mouse anti-NeuN antibody (1:300, MAB377, Millipore, Bedford, MA, USA) at least overnight at 4°C. The L4 DRG sections were incubated with rabbit anti-mouse TRPV1 antibody (1:300) and rabbit anti-CGRP antibody (1:5000) overnight at 4°C. Spinal cord sections were rinsed with PBS three times and incubated with a mixture of secondary antibodies containing Alexa Fluor 488-labeled goat anti-rabbit IgG (1:200, A11001, Molecular Probes, Eugene, OR, USA) or Alexa Fluor 594-labeled goat anti-mouse IgG (1:200, A11012, Molecular Probes) for 2 h at room temperature. The DRG sections were incubated with Alexa Fluor 594-labeled goat anti-rabbit IgG or Alexa Fluor 488-labeled goat anti-rabbit IgG as a secondary antibody. After being washed three times in PBS, the sections were examined under a fluorescence microscope. The TRPV1- and CGRP-positive DRG neurons were counted manually on captured images and expressed as the percentage of total DRG neurons. Quantitative analysis of TRPV1-positive neurons in spinal cord was performed by using NIH ImageJ software as described previously [63]. Briefly, the outline of the superficial dorsal horn was manually traced for each image, and then an appropriate threshold was set such that only specific TRPV1-immunoreactivity was accurately represented and light nonspecific background labeling was not detected. The threshold was the same for all images. The density limited to threshold in the outlined area was measured. The TRPV1-immunopositive density was calculated for each image.

The pCREB immunohistochemical analysis was performed using lumbar spinal cord samples collected from mice sacrificed at 20 min following the formalin injection as described previously [22]. The endogenous peroxidase was quenched by incubating the samples in PBS with 0.3% hydrogen peroxide for 30 min. Sections were then incubated with a rabbit p-S133 rabbit monoclonal antibody for pCREB (1:400, Cell Signaling Technology, Danvers, MA, USA) containing 0.25% normal goat serum overnight at 4°C. The sections were incubated with biotinylated goat anti-rabbit IgG (1:200, Vector Laboratories, Burlingame, CA, USA) at room temperature for 1 h and then incubated in Vectastain Elite ABC Reagent (PK-6101, Vector Laboratories) for 1 h followed by incubation in a diaminobenzidine solution (ImmPACT™ DAB substrate, SK-4105, Vector Laboratories) for 1 min. Sections were washed in PBS for 45 min between each step. The slides were coverslipped with VectaMount AQ (Vector Laboratories) and examined under a light microscope, and images were captured under 100× magnification. Neurons positive for pCREB in the dorsal horn of the spinal cord gray matter were manually counted on the captured images.

### The effects of an LTB_4_ receptor antagonist

ONO-4057 (supplied by Ono Pharmaceutical Co., Osaka, Japan) is a potential BLT1 antagonist and permeability of Blood Brain Barrier is not clear [64,65]. ONO-4057 was dissolved in a 2.1% sodium bicarbonate solution. Intraperitoneal (0.125 or 0.25 mg/mouse, 250 μL volume) and intraplantar (0.014 or 2.35 μg 10 μL volume) ONO-4057 injections were administered 45 min prior to the formalin intraplantar injection. Intrathecal injections of ONO-4057 (10 or 20 μg/mouse, 5 μL volume) were administered 30 min prior to the formalin injection, as described previously [66]. Briefly, mice were shaved on their lower backs, placed in nose cones and injected between the L5 and L6 vertebrae using a 25-μL microsyringe with a 30-gauge needle. Monitoring the tail-flick response of the animal assessed the success of the intrathecal injection. Intraperitoneal and intraplantar doses of ONO-4057 were selected based on previous reports [67,68]. The control mice were pretreated with vehicle (2.1% sodium bicarbonate solution) before formalin intraplantar injections.

### Statistical analysis

All results are expressed as mean ± SEM. Two-way analysis of variance (ANOVA) and Bonferroni post hoc tests were applied to the analysis of the behavioral data, MPO activity measurements and to the pCREB immunohistochemical analysis (for comparisons between BLT1WT and BLT1 KO mice). Assessment of paw edema, qRT-PCR, analysis for the immunohistochemical profiles of nociceptive neurons and pCREB immunohistochemistry (ONO-4057 pretreated mice) were conducted using unpaired Student’s t-tests with Welch’s correction. Plasma extravasation data were analyzed using a Kruskal-Wallis test followed by Dunn post hoc test. The criterion for statistical significance was p < 0.05.
